# Scale of Small Particle Population in Activated Sludge Flocs

**DOI:** 10.1007/s11270-018-3979-7

**Published:** 2018-09-24

**Authors:** Magdalena Kuśnierz

**Affiliations:** 1 Wroclaw University of Environmental and Life Sciences, Wroclaw, Poland; 2 Institute of Environmental Engineering, Plac Grunwaldzki 24, 50-363 Wroclaw, Poland

**Keywords:** Sludge flocs, Fractal dimension, Particle size distribution, Flocs size, Light scattering

## Abstract

The light scattering method is a valuable tool for accessing particle size and structure mainly due to fast and the nonintrusive nature of the measurement. The method is based on a scattered intensity pattern and depends on particle volume, particle morphology, the light wavelength and the scattering angle. The light scattering model, for particles characterised by a fractal structure, is enabled with the use of the Rayleigh-Gans-Debye theory under constrained assumptions. The range of validity of the Rayleigh-Gans-Debye is limited when primary particles constituting aggregate have a size close to the wavelength. In this work, a range of particle sizes was characterised in order to achieve a better understanding of the relationship between flocs size and its fractal dimension. Hence, the width of the power law regime is discussed. What is more, a specific fractal dimension value of activated sludge flocs was found for each of the analysed wastewater treatment plant, which suggests that the spatial structure of suspensions constituting the activated sludge is an individual characteristic of each treatment facility. It has been shown that activated sludge consists of microflocs from the range of 1–10 μm, which constitute approximately 90% of all the population.

## Introduction

The geometric characteristics of activated sludge flocs have become the subject of intensive research in the past decades. Currently, it is widely accepted that fractal structure and particle size of flocs are decisive properties having a great impact on the wastewater treatment efficiency (Zheng et al. 2011; Zhao et al. 2013; Zheng and Wang 2017). The structure of the flocs is quantified in terms of fractal dimension (*FD*) with the value 1–3. Neither the *FD* value nor the activated sludge flocs size is a constant value, since they change with the condition changes in the processes of wastewater treatment (Xie et al. 2017).

The structure of activated sludge flocs is intrinsically connected to size (Masse et al. 2006). Fractal aggregates become denser as they increase in size. They usually have a complex structure, with different compaction at different scales and affect surface morphology (Mei et al. 2016). Additionally, the extracellular polymeric substance (*EPS*) of flocs is also a key factor that influences the activated sludge structure and size (Li et al. 2016; Yuan et al. 2017; Zhang et al. 2018). Thus, *FD* may vary considerably, and it is difficult to describe the floc structural configuration. However, Whitehouse (2001) or Ibaseta and Biscans (2010) stated that the FD value obtained in various research may be determined to a greater extent by the instrument capacity than by the actual geometry. The size of flocs and the *FD* values are determined with use of various measurement techniques and analytical methods (Bushell et al. 2002). Vahedi and Gorczyca (2012) distinguish between direct and indirect methods for the determination of *FDs.* As far as measurement techniques are concerned, the oldest applied method was the analysis of the images provided by high quality cameras, electron, optical and confocal microscopes. Although this measurement method has numerous advantages, the compacted mass of flocs sometimes prevents deeper observation of the structure and it may result in the fact that FDs > 2 are underestimated by 10–20%, compared to other approaches that are sensitive to three dimensional structure (Rahmani et al. 2005). The techniques sensitive to three-dimensional structures are considered to be light diffraction and light scattering techniques used in modern analysers. In these devices, the analysis of the light scattering intensity at different angles with respect to the optical axis of the light source allows to evaluate floc size and to determine the optical *FD* (Thill et al. 2000). The light scattering model for particles characterised by a fractal structure is enabled with the use of the Rayleigh-Gans-Debye theory (RGD). This theory assumes that the total scattered wave is the vector sum of the scattered waves from all primary particles (Wu et al. 2002) and the *RGD* model is valid for particles of low refractive index and an appropriate ratio of particle size to the light wavelength. Thus, the representation is correct provided that the aggregates are smaller than the wavelength of the incoming light and provided that the phenomenon of multiple scattering of the light wave vector on particles is excluded. Hence, *FDs* are determined not for all but only for a selected group of particles constituting the activated sludge.

During research on the activated sludge structure with use of laser diffraction, the author focused in particular on the range of particle sizes for which it is possible to determine *FDs*. It should be mentioned here that the discussed research did not involve the issue of oxygenated granulated activated sludge. Sludge granulation is forced by certain technological conditions of the reactors, which were, however, not fulfilled in the analysed treatment plants. Tests were conducted at four facilities. Selecting several wastewater treatment plants (WWTP) located in different towns enabled to obtain practical results of the tests of activated sludge suspension, independent from the type of sewage system, concentration of pollutants in wastewater, the presence of the industrial waste and others.

The aim of the present study is to help establish whether the particle size range within fractal dimensions that can be determined is regulated by measurement capabilities of the laser granulometer and the common Rayleigh approximation limits and whether it includes only particles smaller than the wavelength of the incoming light. So far, none of the research papers have provided precise information about the sizes of particles/flocs, for which it is possible to determine *FD* with use of laser diffraction. Due to the fact that the fractal structure of flocs has a great impact on the wastewater treatment processes, the new approach could be a great support thereto. This work will also be helpful in improving the presence of an enormous number of micron-diameter particles, which might affect the processes related to wastewater treatment.

## Material and Methods

### Scope of the Research

The tests were conducted on activated sludge samples collected from four mechanical-biological *WWTPs* located in the Lower Silesian region in Poland, in the towns: Kąty Wrocławskie, Kobierzyce, Siechnice and Sobótka. Three of the discussed *WWTPs* were operating as flow reactors (Kąty Wrocławskie, Kobierzyce and Sobótka), and one was operating as a sequencing batch reactor (Siechnice). The common elements for all *WWTPs* were screens, grit chambers and primary settling tanks. Secondary settling tanks were present at the *WWTPs* in Kąty Wrocławskie, Kobierzyce and Sobótka. The analysed *WWTPs* were characterised by the flow capacity ranging from 300 to 2740 m^3^/day and varied technological arrangements. The selected locations were a small distance away from each other to enable sample’s collection on the same day.

Activated sludge samples were collected in the years 2008–2010 and 2014–2015, at 1-month or 2-month intervals, both during the summer and winter season. However, no significant correlations were found between the granulometric composition of suspensions collected in the same seasons of the year. During 3 years, a total of 69 activated sludge samples obtained from four *WWTPs* were analysed. One sample of activated sludge of a volume of 1.5 l was collected from each *WWTP* on each given day. During the whole research period, samples were collected from the same sites, directly from the activated sludge reactors in the nitrification phase, with use of a scoop mounted on an arm. The material was collected each time, from all facilities, between 8.00 a.m. and 12.00 p.m., and the period between collecting samples and analysing them in laboratory did not exceed 4 h.

### Particle Size Analysis

The analyses of particle size distribution (*PSD*) were performed with use of Mastersizer 2000 laser granulometer. This instrument is equipped with an optical system consisting of red and blue light sources, a set of detectors and a measurement cell. The light source is a helium-neon laser of a wavelength of 632.8 nm. Three sets of appropriately placed detectors allow for the measurement of the scattered light intensity nearly in the full range of angles. The central detector enables the identification of large particles, side detectors are used for the determination of micro-suspension size and reverse detectors identify colloidal particles. The software enables to calculate particle size within the range from 0.02 to 2000 μm and to determine the particle number *n*_i_ and particle volume *v*_i_ distributions (*f*(*n*_i_) and *f*(*v*_i_)) of diameter *d*_*i*_ basing on the light dispersion patterns on suspended particles recorded by the optical unit, according to Mie theory.

Activated sludge samples were analysed with use of the manual wet dispersion unit HYDRO MU which is an element of the Mastersizer. In order to obtain the correct quality of measurement data, it was necessary to prepare activated sludge samples containing an appropriate concentration of suspensions so that the obscuration would fall into the range of 10–30%. If particles are properly dispersed in the measurement chamber, it allows avoiding multiple scattering that occurs during the evaluation of *PSD* in suspension sets with a large solid fraction. The content of activated sludge solid suspension determined during separate tests ranged from 2 to 10 mg/l. Thus, it was necessary to reduce the concentration in order to obtain correct measurement results. The *PSD* for each sample was obtained based on several replications of one measurement. A single measurement consisted of 15 repetitions lasting for over 10 s, taken at millisecond intervals.

### Particle Size Image Analysis

Optical observations of flocs were made with the use of Malvern Morphologi G3 which applies the technique of automated static image analysis*.* The Morphologi G3 provides particle characterisation tool for the measurement of particle size and particle shape from 0.5 μm to several millimetres. The measurement system is equipped with a *Nikon CFI 60* optical unit and a *CCD* digital camera. The tests of activated sludge started with the preparation and placing a small portion of sample on the wet cell plate (180 × 110 mm) for wet measurements and setting up the analysis with use of Standard Operating Procedure (*SOP*). The adjustment in the SOP tab included, among others, choosing the correct lighting, lens, focus and the size of the scan area. After the initial adjustments were approved, the scanning of the required area began. Depending on the SOP settings (mostly scan area), the measurement can last from 2 min to over an hour. During scanning, the image of each particle is recorded in the software of the instrument, which enables to provide a complete, detailed description of the morphological properties of activated sludge flocs later.

### Fractal Dimensions

The *FD* was calculated from the raw light scattering data according to the method by Guan et al. (1998). *FD* values were determined with use of linear regression. Linear regression estimation error was analysed by determining the confidence level for regression coefficients which corresponded to the *FDs* of the analysed suspensions. The confidence levels were determined with the use of Student’s *t*-distribution, at the 95% confidence interval with this data. The obtained *FD* estimation errors for all activated sludge samples did not exceed the value of ± 0.05. Table 1 contains sample *FD* values along with ranges of estimation errors, while in the further sections of the paper, only the most probable *FD* values are discussed, without providing information about errors. Statistical analyses based on the obtained test results were conducted with use of Statistica software.

**Table 1:** Sample *FD* values determined for selected activated sludge samples along with ranges of estimation errors

WWTP	1	2	3	4
Kąty Wrocławskie	2.28 ± 0.026	2.30 ± 0.040	2.21 ± 0.013	2.25 ± 0.028
Kobierzyce	2.20 ± 0.009	2.20 ± 0.015	2.29 ± 0.030	2.32 ± 0.028
Siechnice	2.07 ± 0.010	2.15 ± 0.012	2.10 ± 0.008	2.18 ± 0.010
Sobótka	2.19 ± 0.024	2.09 ± 0.022	2.21 ± 0.011	2.20 ± 0.011

## Results and Discussion

### Particle Size Distribution

**Fig. 1:**
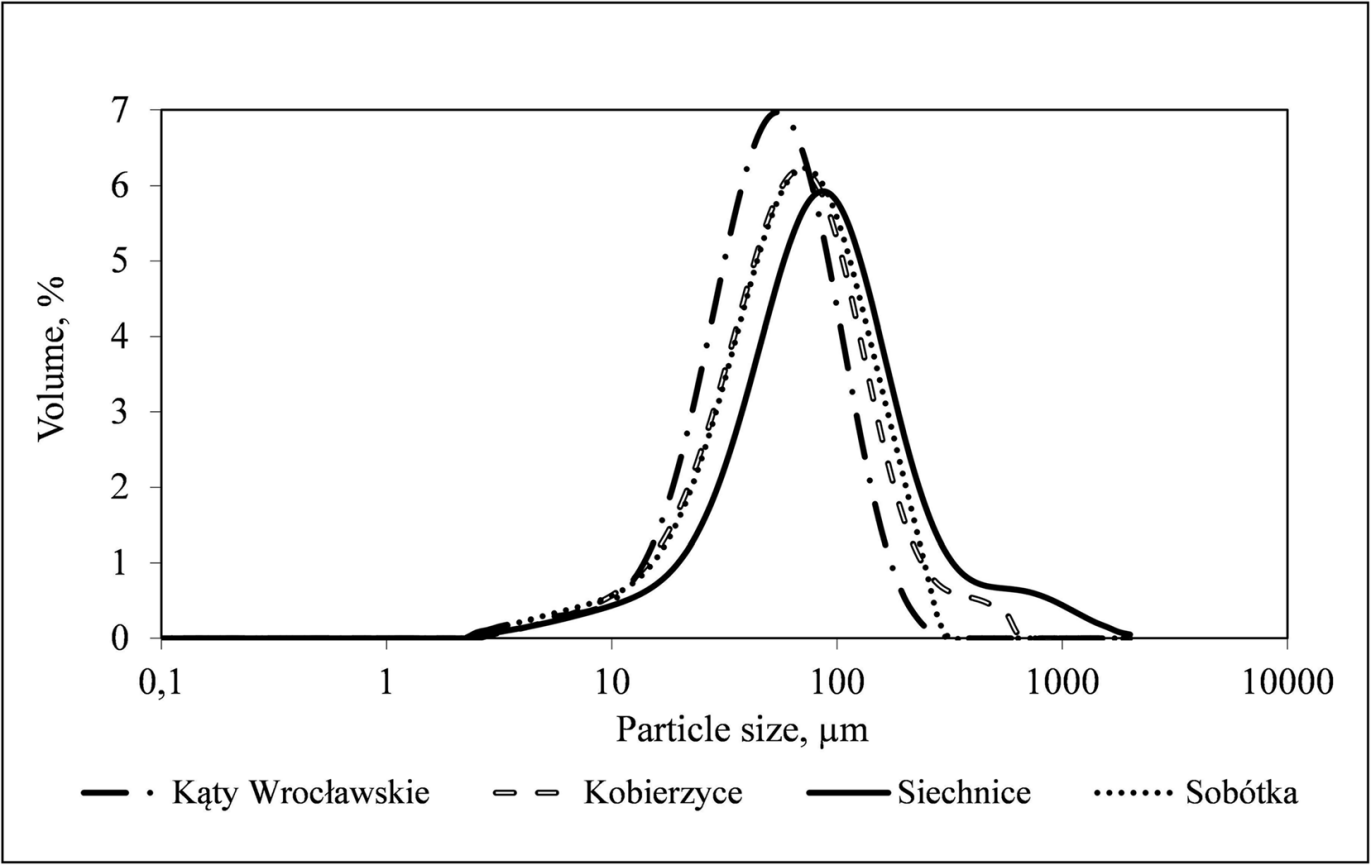
Particle size distribution of *d*_*i*_ diameter in the total volume of activated sludge sample collected from individual *WWTPs*

**Fig. 2:**
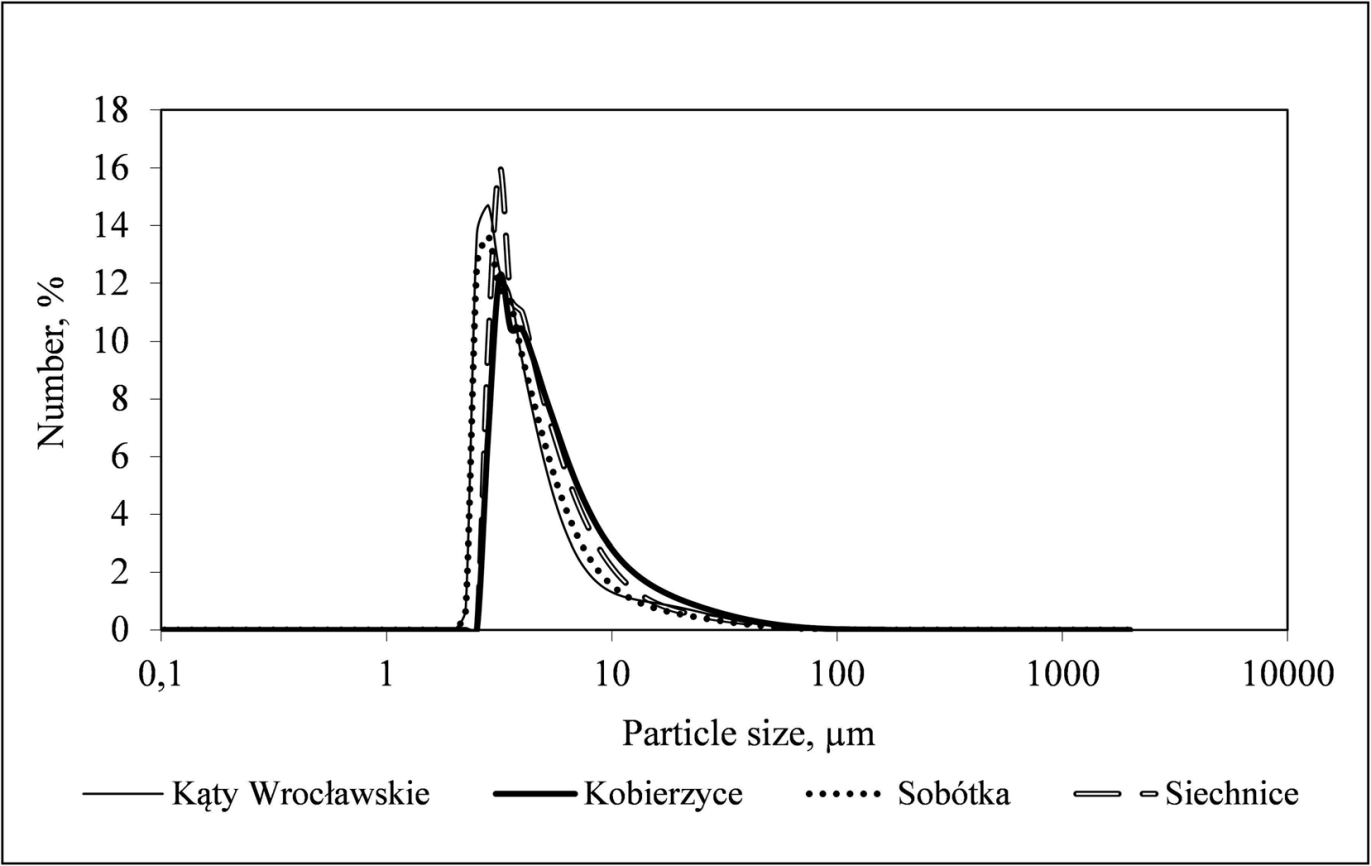
Particle size distribution of *d*_*i*_ diameter in the total number of activated sludge sample collected from individual *WWTPs*

Sample *PSDs* for activated sludge are presented with the use of the *f*(*v*_i_) function in Fig. 1. Figure 2 presents the distributions illustrated with the form of the *f*(*n*_i_) function. The distribution of particle density determined according to particle volume and number differed significantly. Particles of the size from 40 to 200 μm represented the highest volume share, while the smallest identified particles fell into the range from 0.40 to 2.9 μm. For distributions determined based on the particles number, the highest share had particles of the size from 2 to 10 μm. The smallest identified particles of single bacterial cell size, were in the range from 0.36 to 3.56 μm. The volume and number distribution in the particle size ranges below and over 10 μm reveal significant differences in the structure of activated sludge flocs. For the *f*(*v*_i_) distributions presented in Fig. 1, the volume of over 96% of the whole population of particles falls into the particle size range exceeding 10 μm, while only 2.7–4% are in the range below 10 μm. At the same time, for the *f*(*n*_i_) distributions presented in Fig. 2, the range below 10 μm contains from 85.5 to 92.4% of the particle population, and the range above 10 μm contains 7.6–14.4%. This means that most of the flocs constituting activated sludge suspension are very small flocs that are present in large amounts and at the same time have a low mass. Large diameter particles determined percentage values, while the small ones are usually ignored. Similar results were obtained from optical observation of flocs with the use of the Morphologi G3. Selected images of activated sludge flocs, divided according to their size into the ranges > 10 μm, 10–100 μm and 100 μm, obtained for the sample of activated sludge collected from the *WWTP* in Sobótka, are presented in Table 2. On the surface of the wet cell plate during a single measurement with use of the G3 analyser, 72,776 particles were classified and identified, of which 63,658 were microflocs and primary particles of a diameter below 10 μm, while 8985 were flocs between 10 and 100 μm and 8088 flocs above 100 μm. Diameter minimum and diameter maximum were determined as 1.04 μm and 245.72 μm.

**Table 2:**
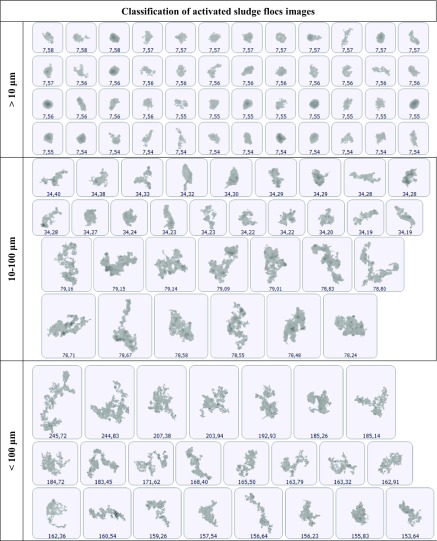
Individual 2D images of activated sludge flocs

### Fractal Dimensions

**Fig. 3:**
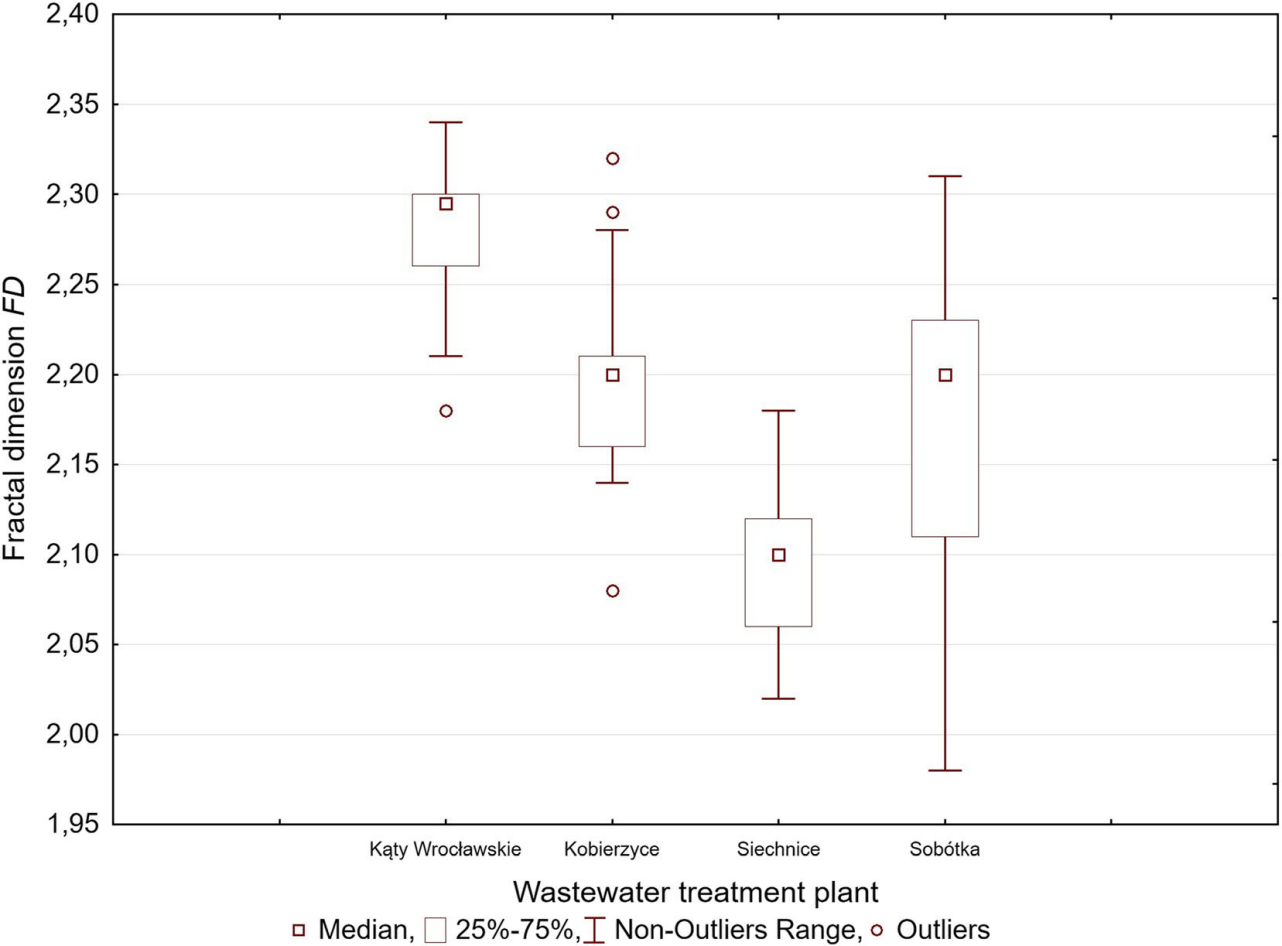
Comparison of the FD values of activated sludge flocs obtained for samples collected from individual WWTPs

The determination of *FD* values was based on the correlation between the light scattering intensity *I*(*θ*) on the scattering angle *θ*, according to the description in Sect. 2.3. Figure 3 shows a comparison of *FD* values for activated sludge flocs presented in the form of the median, quartiles 25% and 75%, and the range of minimum and maximum values. The *FD* values obtained during the test period exceeded 2 (2.02–2.34), with one exception for *WWTP* in Sobótka (1.98). The differences between *FD* values obtained from specific *WWTPs* suggest that the spatial structure of activated sludge flocs is an individual characteristic of the given *WWTP*. The flocs were characterised by a high degree of compaction, and compact flocs have been found to have higher *FD* values.

Studies by Sorensen et al. (1995) have demonstrated that three characteristic regions may be distinguished in the log-log plot of the scattering intensity versus *q*. For the value of the wave vector that meets the condition *q* < < 1, the scattering intensity meets the dependence:

1 $$ I(q)\propto \left(1-\frac{1}{3}{\left({R}_g\right)}^2\right) $$

This region is called the Guinier regime. If *q* > > 1, Prod’s law concerning the phenomenon of light scattering on the surface of primary particles applies:

2 $$ I(q)\propto {q}^{-4} $$

For the value of the wave vector *q* in the range:

3 $$ \frac{1}{a}\le q\le \frac{1}{R_{\mathrm{p}}} $$

where *R*_p_ is the primary particle size that is identified by scattering light of wavelength *λ*, while *a* is the largest particle that does not cause light scattering described by Prod’s law; the following dependence exists:

4 $$ I(q)\propto {q}^{-{D}_f} $$

Analysing the changes of scattering intensity *I*(*q*), it is possible to determine the value of the optical *FD* and the limiting values of particle sizes described by this *FD*. The *FD* can be determined from Eq. 4 for particle size that meets the following condition (Eq. 5) (Spicer et al. 1998).

5 $$ {d}_o\ll \lambda $$

During the analyses conducted with the use of a laser granulometer, it was noted that in the (*R*_p_^−1^; *a*^−1^) range, there is a possibility to convert the wavenumber *q* into the particle diameter *d* denoted in micrometer. So far, none of the research papers have provided precise information about the sizes of particles/flocs, for which it is possible to determine *FD* with use of laser diffraction.

**Fig. 4:**
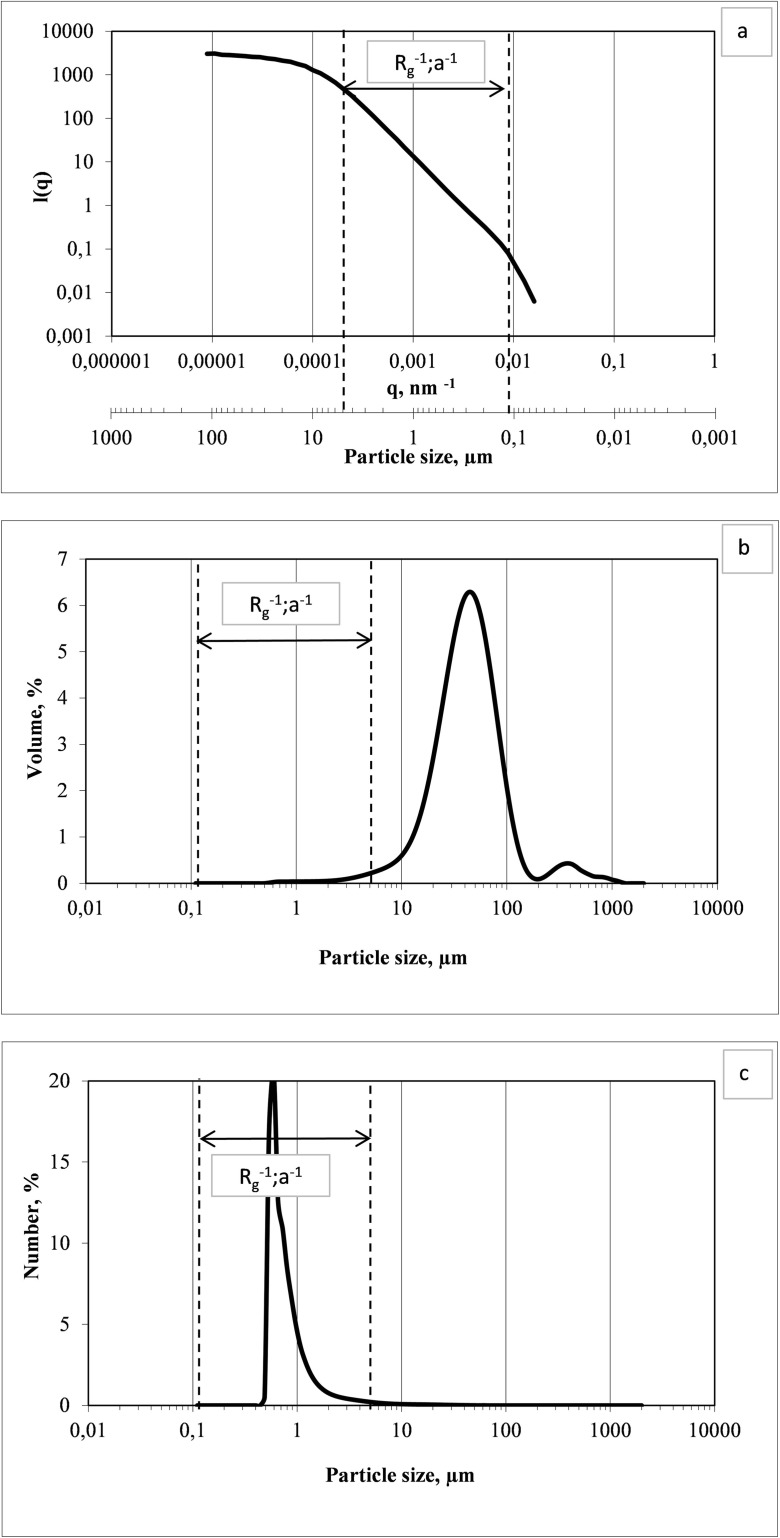
Determination of FD with marked particle size range (**a**), particle size distribution of *d*_*i*_ diameter in the total volume (**b**) and number (**c**) of activated sludge collected from the WWTP in Kąty Wrocławskie

Figure 4a shows the course of the *I*(*q*) function that is used for the determination of *FD* for flocs of activated sludge originating from *WWTP* in Kąty Wrocławskie. Additional lines in the figures mark the limits of the (*R*_p_^−1^; *a*^−1^) range, which, at the same time, define the minimum and maximum values of *q* and *d*. The delimiting lines were then transferred to the diagram of the particle volume and number distribution (Fig. 4b, c). As it was mentioned in Sect. 3.1, the range of small size particles is hardly noticeable in the particle volume distributions (Fig. 4b). Percentage share values are determined by large size particles, while small size ones are completely neglected. Figure 4c illustrates size values identified in the region (*R*_p_^−1^; *a*^−1^), for which *FDs* were determined and which corresponded to the size of particles that had the highest percentage share in particle number.

**Fig. 5:**
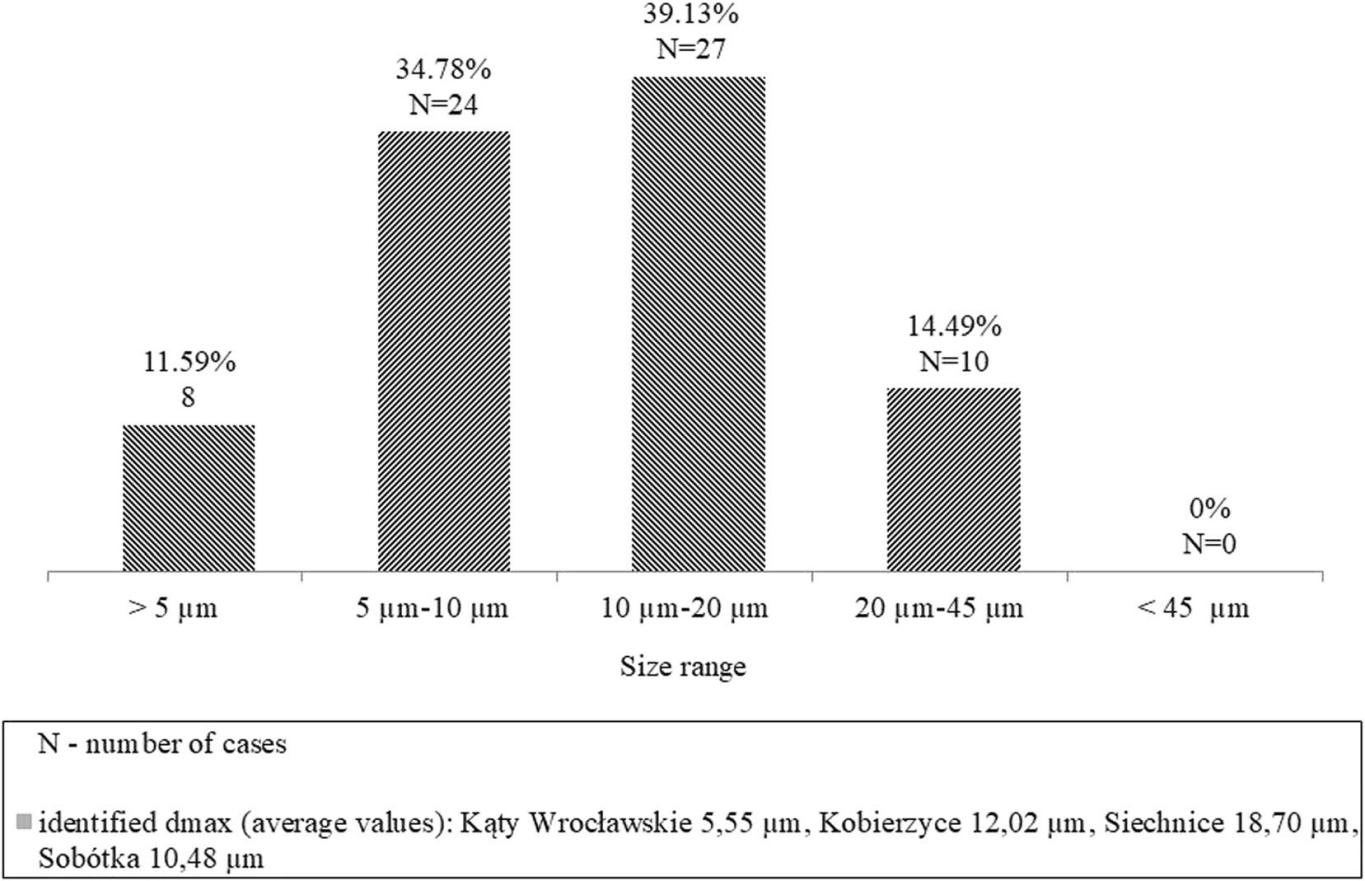
The maximum values of floc size that fell into the (*R*_p_^−1^; *a*^−1^) range (presented for all 69 measurements)

Flocs size determined in the (*R*_p_^−1^; *a*^−1^) range was calculated for samples collected from all *WWTPs.* Minimum values of floc size that fell into the (*R*_p_^−1^; *a*^−1^) range were below 1 μm. As far as maximum diameters are concerned, most flocs fell into the 10–20 μm range (39.13%), and the fewest ones in the 4.5–5 μm range (11.59%) (Fig. 5). The largest range of flocs sizes determined in the (*R*_p_^−1^; *a*^−1^) range was noted for samples collected from *WWTP* in Siechnice, where, at the same time, two extreme values for the whole data set were noted: 30 μm and 45 μm. The narrowest range of flocs size was found in sludge collected from *WWTP* in Kąty Wrocławskie, for which the highest *FD* values were noted.

## Discussion

The size and spatial structure of activated sludge flocs are important properties that influence the effectiveness of the wastewater treatment process. Flocs which are too small or too large do not perform properly their task connected with wastewater treatment. Raynaud et al. (2012) pointed out that large sludge flocs are characterised by slower sludge filterability and dewaterability of flocs results from the influence of fine particles. On the other hand, medium-size and small flocs have good access to oxygen and pollutants being a nutrient. Han et al. (2012) discovered that with floc particle size increasing from 100 to 250 μm, the dissolved oxygen (*DO*) concentrations in the floc centres decreased by 10–55%. Although large activated sludge flocs sediment well, they treat wastewater poorly. However, in large flocs with particle sizes of more than 100 μm, bacterial diversity was found to be abundant than those with the particle size of less than 100 μm (Han et al. 2012). Thus, it can be assumed that activated sludge flocs of different sizes are beneficial for the removal of pollutants in *WWTPs*. In the analysed *WWTPs*, the size of activated sludge flocs ranged from 0.35 to 2000 μm. Particles of the diameters from 40 to 200 μm had the highest percentage share in particle volume, while particles of diameters below 10 μm had the highest share in their number. The range below 10 μm accounted for 85.5% to 92.4% of the total particle number. Similar results concerning the particle sizes in activated sludge, of approximately 300 μm, were obtained by Defrance et al. (2000), while the values found by Wisniewski and Grasmick (1998) exceeded 500 μm. On the other hand, Wang et al. (2014) obtained a distribution curve with a mean representative diameter (*d50*) of about 100 μm from the granulometric analysis of sludge.

The dominant *PSDs* obtained for the analysed activated sludge samples were onemodal with the peak of particle size around 100 μm. However, the concealed group in volume distributions are microflocs from the 2–10 μm range. Onemodal *PSDs* for activated sludge were also observed by Gonze et al. (2003) and Chaignon et al. (2002), while the existence of bimodal systems was proven, among others, by Lower (quot. Houghton et al. 2002). Wu et al. (2009) also pointed out that activated sludge is characterised by a bimodal *PSD* with one group in the range of 0.5–5 μm and another group in 25–2000 μm range. Flocs with particle size in the range of 0–250 μm formed the major part of activated sludge, and those below 250 μm were investigated as they dominated in flocs. Li and Ganczarczyk (1990) confirmed the presence of microflocs of diameters lower than 5 μm in sludge, with a significant volume occupied by flocs of sizes in the 68–183 μm range. On the other hand, Jordan et al. (1995) pointed out the presence of particles of a diameter of 2.5 μm in the zone of sludge flocs of 125 μm size. This means that activated sludge flocs are organised on different structural levels.

According to Wu et al. (2002), the primary particles that build activated sludge flocs are mainly of a multi-fractal nature. Due to that, the evaluation of *FD* in the tests of aggregates composed of the same particles, monodispersive in terms of both shape and size (Bushell 2005; Spicer et al. 1998; Thill et al. 2000) is a major simplification performed by the authors. Additionally, the *FD* value is not constant for the given type of suspensions and it changes depending on the conditions of the wastewater treatment process.

In this experiment, the obtained *FD* values fell into the 1.99–2.31 range. Similar *FD* values for activated sludge flocs were obtained, among others, by Waite (1999), Masse et al. (2006) and Mei et al. (2016). The *FD* values were determined based on the log-log plot of the scattering intensity versus *q* in the range (*R*_p_^−1^; *a*^−1^). The conducted research demonstrates that *FD* describes the spatial structure of flocs that are dominant in terms of number in the activated sludge and form the lowest organisational level of floc. For example, the *FD* value obtained for activated sludge floc equal to 2.26 corresponded to structure consisting of microflocs and primary particles of sizes up to 30 μm. At the same time, it was determined that the lower value of the *FD* was calculated for particles from the wide size range within the (*R*_p_^−1^; *a*^−1^) limits. This means that the ranges presented on Fig. 5 exceed the limits defined by the *RGD* approximation and relate to particles constituting flocs with a size larger than the laser wavelength as well. Much earlier, studies by Bushell et al. (2002) pointed out that the *RGD* law seemed to be too conservative as far as the size of particles that may be described by *FD* values is concerned. The existing literature shows that the static light scattering method has been successfully applied to characterise aggregates of small particles. However, Spicer et al. (1998) mentioned that it can be used to evaluate the structure of kaolin composed of particles larger than the laser wavelength. Thus, one may conclude that it is required to introduce several measurement methods to fully evaluate the structure of activated sludge flocs characterised by a multi-fractal structure. Laser diffractometers seem to have become a less attractive measurement tool than other optical methods since only one averaged fractal dimension is obtained even for complex flocs. However, laser diffraction will be helpful in evaluating the internal structure and *FD* of micro flocs, while image analysis is much more suitable for evaluating the outer structure of the flocs (Chen and Wang 2015) and *FD* of macro-flocs of the sludge, which was, by the way, suggested by Ibaseta and Biscans (2010).

## Conclusions

In this study, size and *FDs* of activated sludge were determined from laser diffraction. Sensitive to three-dimensional structure method, such as laser diffractometry, proves to be quite reliable in floc size measurements and, under certain assumptions, floc geometry. Laser diffraction will be helpful in evaluating the *FDs* of selected groups of flocs that are dominant on *f*(*n*_i_) distributions. A specific *FD* values of activated sludge flocs was found for each of the analysed *WWTPs*, which suggests that the spatial structure of suspensions constituting the activated sludge is an individual characteristic of each treatment facility. The *FD* value obtained for 69 activated sludge samples corresponded to a structure consisting of microflocs and primary particles of sizes up to 45 μm. Importantly, this research has shown that in the power law regime (*R*_p_^−1^; *a*^−1^), a wider range of particle sizes may be identified with *FD*s than it is foreseen by the *RGD* law.

As far as the size of activated sludge flocs is concerned, it may be realistically assessed by laser diffraction in the whole measurement range. Distributions based on the particles volume had the highest share of particles with the size from 40 to 200 μm. However, particles of the size from 2 to 10 μm represented the highest share on particle distributions. This means that most of the flocs constituting activated sludge suspension are very small flocs that are present in large amounts and at the same time have a low mass. The presence of an enormous number of micron-diameter particles might affect processes related to wastewater treatment.
